# Salivary cortisol levels in patients with potentially malignant oral disorders and oral mucosal cancer: a case-control study

**DOI:** 10.4317/medoral.26606

**Published:** 2024-05-25

**Authors:** Edgardo López-D’alessandro, Judith Palomino, Livia Escovich

**Affiliations:** 1 Chair of Clinical Stomatology II, School of Dentistry, National University of Rosario, Argentina. Dentist, Bachelor’s Degree in Dentistry, Master in Stomatology-Oral Medicine, Master in New Trends in Health Sciences Research, Doctor in Dentistry; 2 Chair of Clinical Stomatology I, School of Dentistry, University of Rosario, Argentina. Dentist; 3 Ex Professor of Clinical Stomatology, School of Dentistry, University of Rosario, Argentina

## Abstract

**Background:**

The relationship between salivary cortisol secretion and the presence of cancer in the oral cavity has not been completely clarified. Due to this, we proposed carrying out a study to determine salivary cortisol levels in patients with potentially malignant disorders (PMD), oral squamous cell carcinoma (OSCC) and healthy individuals.

**Material and Methods:**

Cross-sectional case-control study, among 80 patients seen at the Faculty of Dentistry of the National University of Rosario, Argentina, between January 2018 and April 2020. 40 cases represented by Leukoplakia, Lichen, Erythroplakia and SCC and 40 controls were included. Smoking habit and alcohol consumption were included. The presence of stress was determined. Morning salivary cortisol levels were measured with the Roche electrochemiluminescence method (Traceability: St by ID-MS).

**Results:**

Patients with SCC presented elevated salivary cortisol values. Individuals with stage III tumors showed levels higher than 8.74 ng/ml in all cases. A significant association between cortisol levels and stress was detected in patients in the control group (*p*<0.005) and in individuals with PMD (*p*=0.009). This association was not significant in patients with SCC (*p*=0.999). After applying the logistic regression method, when adjusting odds ratios according to tobacco and alcohol consumption and the presence of stress, the association between cortisol levels and presence of stress was highly significant (*p*<0.001). The possibility of presenting undetecTable cortisol results was 94% lower in patients with stress.

**Conclusions:**

The increase in salivary cortisol levels in patients with PMD and SCC, is related to stress conditions, being able to generate alterations tending to immunosuppression of the cellular microenvironment.

** Key words:**Salivary cortisol levels, oral cancer, potentially malignant disorders, psychosocial stress.

## Introduction

Cortisol is the glucocorticoid hormone produced by the adrenal cortex, which helps the body to use glucose, proteins and fats. It is also known as the stress hormone, since our body produces it in greater quantities in emergency situations. The secretion of cortisol is determined by psycho-neuro-immunological factors inherent to the individual, although its rate can be variable. Cortisol release is regulated by the hypothalamus and occurs at low blood glucose levels or in response to the presence of stress, with chronic stress being the main external factor responsible for the prolonged increase in cortisol levels. Dysregulation of the hypothalamic-pituitary-adrenal axis generated by situations of stress, anxiety or depression, produces increased cortisol levels that have been related to the appearance or aggravation of different types of cancer. There is a proven correlation between increased cortisol levels and tumor progression in malignant tumors of the head and neck (1). On the other hand, there is a relationship between increased cortisol levels and advanced stages of oral cancer (2). Tumor progression is the result of interaction within the tumor and with its surrounding supporting tissue, which can be influenced by the tumor microenvironment (3). Oral mucosal cancer is a disease that can occur in healthy mucosa, or develop in areas with previous alterations called potentially malignant oral disorders (OPMDs), formerly known as precancerous lesions or conditions. The WHO defines OPMDs as any abnormality of the oral mucosa that is associated with a statistically increased risk of developing oral cancer (4). The prevalence of OPMDs is highly variable with estimated rates ranging from 4.47%, comprising 0.11% in North American populations to 10.54% in Asian populations (5-6). Patients diagnosed with OPMD are more susceptible to develop cancer in their oral cavity during their lifetime. Moderate/severe dysplasia carries a much higher risk of cancer progression than mild dysplasia. It is important to raise awareness of the malignant transformation rates of these disorders, which along with strict follow-up measures and optimal treatment strategies would help reduce the transformation of these oral conditions into invasive cancer (7). The possibility of malignant transformation of these disorders is variable. In Argentina, between 3 and 7% of detected potentially malignant oral mucosal disorders develop into cancer. Among the most frequent OPMD are Leukoplakia, Erythroplakia and Oral Lichen Planus. Worldwide, Leukoplakia has a malignant transformation rate of 3% for homogeneous lesions and 14.5% for non-homogeneous lesions (8). Erythroplakia has high malignant transformation rates ranging from 14% to 85% (9). On the other hand, Oral Lichen Planus has a malignant transformation rate of 1.14%, with a higher possibility of malignization in atrophic or erosive lesions, presenting the highest risk when located on the tongue (10). The actual transformation rates in this type of lesions are difficult to calculate, because, lesions with moderate and severe dysplasias are excised upon detection (11). Several authors have observed a dysregulation of the hypothalamic-pituitary-adrenal axis in patients with oral and pharyngeal cancer, with changes in plasma and salivary cortisol levels, relating these increases to psychological symptoms responsible for advanced stages of oral cancer (1,2). On the other hand, there are no studies correlating increases in salivary cortisol with OPMD, nor comparing these levels with those found in SCC. Neither has it been possible to establish the usefulness of this hormone as a biomarker related to the clinical stage progression of SCC, nor the increasing relationship of salivary cortisol levels in OPMD and SCC to date. Because of this, we consider the need to carry out this study that allows us to evaluate the levels of salivary cortisol in patients with Leukoplakia, Erythroplakia, oral lichen planus and SCC and to compare them with a control group; taking into account the different risk factors associated with the appearance of SCC such as the use of tobacco, alcohol and the presence of psychosocial stress.

## Material and Methods

An analytical observational study of Cases and Controls was carried out among patients treated at the Stomatology service of the Faculty of Dentistry of the National University of Rosario, Argentina. They decided to participate voluntarily, in a period between February 1, 2018 and April 30, 2020. The current work was based on previous pilot work, carried out in March 2016. The study was previously approved by the institution's bioethics committee. Patients with Potentially Malignant Oral Lesions, represented by Lichens (atrophic-erosive or keratotic); Leukoplakia and Erythroplakia and patients with ESCC (all primary oral carcinomas - first tumors - in different stages) were considered CASES. Patients without pathology in their oral mucosa, matched by sex and age (healthy volunteers) were considered CONTROLS. All OPMD and SCC diagnosed were confirmed by pathological anatomy. The sample size was determined under the assumption that patients with pathologies (OPMD and SCC) had higher salivary cortisol values than the control group by 8.74 ng/ml or more; taking into account that the standard deviation of salivary cortisol, as indicated by our previous studies, was 57 ng/ml, to reach a significance level of 5% and a power of 90% we included a minimum of 32 Cases and 32 Controls. Finally, a total of 40 cases were included, represented by Leukoplakia *n*=10; Erythroplakia *n*=10; Lichen *n*=10, SCC *n*=10 and 40 controls, in order to prevent future dropouts. They were excluded patients with oral malignant tumors not corresponding to SCC; patients with malignant neoplasms of any type, located outside the stomatological area; patients under chemotherapy or in treatment with immunosuppressive drugs; patients who had used corticosteroids of any type or sex steroids (oral contraceptives) in the last six months; patients with comorbidities affecting cortisol secretion: Obesity; Diabetes; Cushing's Syndrome; Addison's Disease and Adrenal Insufficiency secondary to poor pituitary function.

- Previous preparation: Patients with previous infectious oral diseases of dental or periodontal origin were treated with methods of inactivation and elimination of dental infectious foci and total periodontal disinfection techniques, in order to achieve their control before being included in this study.

- Interventions: A complete Medical Record (MR) specially designed for this study was created, where tobacco consumption, alcohol consumption and the presence of stress were recorded, among other data. To determine the presence of stress in the participants, the "Holmes and Rahe scale" was used, which consists of a questionnaire with a list of 43 stressful life events that contribute to the development of disease (12). To assess salivary cortisol levels, saliva samples were collected from the participants. The collection was performed between 8 and 10 A.M. The participants attended without brushing their teeth or eating any food for one hour before the collection, then they rinsed their mouth with cold water and after a ten-minute relaxation of their oral musculature, they collected 1 cubic cm of saliva in a plastic tube with a screw cap. The saliva samples obtained were labeled and kept refrigerated between 6° and 8°C to be transported to the laboratory where they were stored in ultrafreezing at - 20°C.

- Determination of Salivary Cortisol: For the assessment of morning cortisol levels in the collected saliva, the saliva was thawed, centrifuged and analyzed by Roche electrochemiluminescence immunoassay (standardized traceability against IRMM (Institute for Reference Materials and Measurements) / IFCC-451 (ID-GC/MS, gas chromatography with isotope dilution/mass spectrometry). Instrument: Cobas 8000 Roche Auto Analyzer, Cobas e 801 Immunoassay module.

- Dependent or response variable: Morning salivary cortisol level, classified as undetecTable (less than 0.054 ng/ml), detecTable (over 0.054 ng/ml and less than 8.74 ng/ml) or elevated (8.74 ng/ml or higher).

- Independent or explanatory variables: Group of patients (with SCC, with PMD and healthy control), SCC stage (I, II and III), PMD diagnosis (erythroplakia, leukoplakia or lichen), age (in years), tobacco consumption (Yes, No), alcohol consumption (Yes, No), presence of psychosocial stress (Yes, No).

- Statistical analysis: Quantitative variables were described with frequencies and percentages. Numerical variables were described in range, mean, deviation and quartiles. To compare the numerical variables in the groups, we used the t-student test for independent samples, when the conditions of validity were met. Otherwise, the nonparametric Mann-Whitney test was used. To study the differences in salivary cortisol levels among the four types of pathologies, we used the one-factor ANOVA test when the conditions of validity were met. Otherwise, the nonparametric Kruskal-Wallis test was used. Finally, a logistic regression model was adjusted considering as response variable the morning salivary cortisol dosage (undetecTable or detecTable) and as independent or predictor variables, alcohol consumption, smoking status and presence of stress.

## Results

The baseline characteristics of the individuals under study were evaluated according to the group to which they belonged. No significant differences were found in the distribution of the variables according to the group, with the exception of tobacco consumption, since the percentage of individuals with this habit was higher in patients with SCC compared to those in other groups (*p*=0.037) (Table 1). Only in patients with SCC, salivary cortisol values higher than 8.74 ng/ml were found (30%). In that group, 20% presented undetecTable values (level lower than 0.054 ng/ml), compared to 53.33% observed in patients with PMD and 60% detected in controls. The difference when comparing the percentage of undetecTable cases among SCC patients versus controls was statistically significant (*p*=0.035).

**Figure 1:**
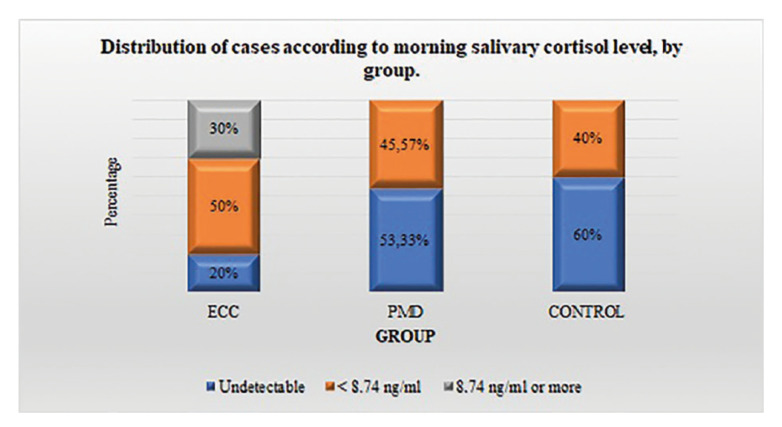
Distribution of cases according to morning salivary cortisol level, by group.

The chance of having an undetecTable result was 85.8% lower in SCC patients than in controls (OR=0.14, 95%CI=0.04;0.93). The chance of having an undetecTable level in individuals with PMD did not differ significantly from that of controls (OR=0.68, 95%CI=0.29;1.97, *p*=0.631) (Fig. 1).

**Figure 2:**
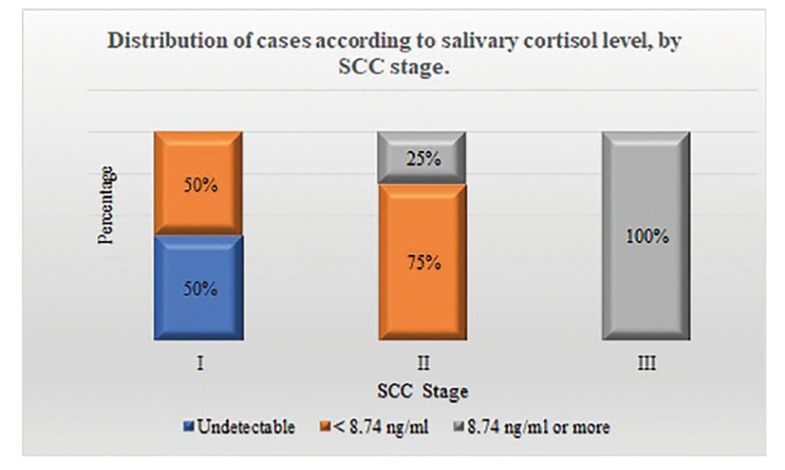
Distribution of cases according to salivary cortisol level, by SCC stage.

Of the 10 SCC patients, 4 (40%) had stage I tumors; 4 (40%) had stage II tumors and 2 (20%) had stage III tumors. Of the individuals with stage I tumors, half had an undetecTable level and half had a level below 8.74 ng/ml. Among patients with stage II SCC there were no undetecTable levels, 3 cases (75%) had values below 8.74 ng/ml and one case had a level above that value. The two individuals with stage III tumors showed a morning salivary cortisol dosage higher than 8.74 ng/ml (Fig. 2).

**Figure 3:**
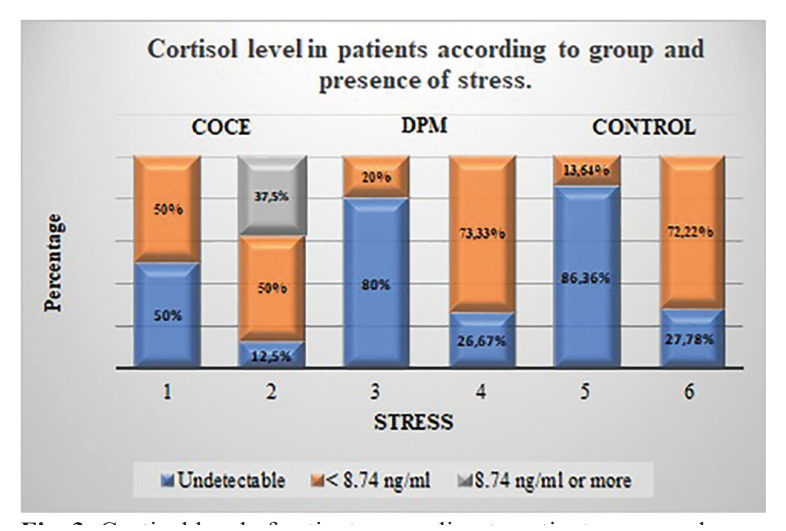
Cortisol level of patients according to patient group and presence of stress.

A significant association between cortisol level and stress was found in patients in the control group (*p*<0.005) and in individuals with PMD (*p*=0.009). This association was not significant in patients with SCC (*p*=0.999) (Fig. 3).

After adjusting the odds ratios of the groups according to tobacco and alcohol consumption and the presence of stress, a significant association was observed between the response variable and alcohol consumption (*p*=0.005). The possibility of presenting an undetecTable cortisol result was 7.21 times higher in patients who consumed alcohol compared to those who did not. In addition, the association between cortisol level and the presence of stress was significant (*p*<0.001). The possibility of presenting an undetecTable cortisol result was 94% lower in patients who presented stress compared to those who did not (Table 2).

## Discussion

Several studies have proved the effect of neurohormonal products derived from chronic stress on cancer progression; however, there is little information on the influence of cortisol on oral cancer development. (12-15) Our study was designed to analyze morning salivary cortisol levels in patients with PMD and SCC and to evaluate their correlation with toxic habits (tobacco and alcohol), taking into account the psychological parameters present in the Holmes and Rahe objective stress scale (also called social readjustment scale or SRRS in these patients) and compare them with healthy patients. In a study by Sharma *et al*. (1), anxiety and depression scale scores were higher in the SCC group compared to the other groups, also showing higher levels of anxiety and depression in these patients, with the mean plasma cortisol level and mean salivary cortisol level in the SCC group being twice as high compared to the mean levels in the control group. In our study, within the SCC group of patients, 80% presented detecTable morning salivary cortisol levels; while in the Lichen group 50%, in the Leukoplakia group 30% and in the Erythroplakia group 60%. Paleri *et al* (16) found that elevated plasma cortisol levels are a constant feature in patients with head and neck cancer from the period of diagnosis until 6 months later. Bernabe *et al* (2) described that plasma and salivary cortisol levels were significantly higher in SCC patients compared with the other groups. Oropharyngeal SCC patients had higher salivary cortisol levels compared with smokers and/or drinkers and patients with leukoplakia. Patients with advanced stage SCC showed higher cortisol levels than those patients with early stage SCC. Men with SCC showed higher salivary cortisol levels than women. In our study, salivary cortisol levels were significantly higher (*p*<0.05) in SCC patients than in healthy patients. No statistically significant differences were found in cortisol levels in PMDs among themselves. Of the individuals with stage I tumors, half had an undetecTable level and half had a level below 8.74 ng/ml. Among patients with stage II SCC there were no undetecTable levels; individuals with stage III tumors all showed a morning salivary cortisol level above 8.74 ng/ml. SCC progression, with reference to stress in oral cancer cell lines has been linked to up-regulated IL-6 production in response to stress hormone. Individuals experiencing emotional stress show high circulating levels of IL-6 (17,18). In our case, the association between cortisol level and the presence of stress was significant; the chance of presenting an undetecTable cortisol result was 94% lower in patients who presented stress compared to those who did not. In recent studies on breast cancer and ovarian cancer (19-21) the experience of cancer in some individuals causes dysregulation of the hypothalamic-pituitary-adrenal axis, with changes in the circadian rhythm of cortisol, which may accelerate tumor progression and reduce quality of life. Cruz *et al* (22) demonstrated that nocturnal cortisol has the best diagnostic accuracy in identifying head and neck cancer patients with poorer quality of life after finding a negative correlation between quality of life and stress levels, as indicated by salivary cortisol concentrations and perceived stress scale score. In our study, the association between cortisol level and the presence of stress was significant (*p*<0.001). At the conclusion of our study we were able to prove that there is a progressive increase in salivary cortisol levels according to the group studied, with lower increases in PMD patients and higher levels in SCC patients. In conclusion, the results obtained would indicate that there is a dysregulation in cortisol secretion in SCC patients, related to the presence of stress, which would favor immunosuppression of the cellular microenvironment. On the other hand, the determination of hormone concentration in saliva has certain advantages over the use of other biological fluids (plasma or urine) because the collection procedure is simple and stress-free. The possibility of obtaining a biological marker represented by the variation in salivary cortisol levels could be useful in the risk of suffering SCC or its aggravation. This fact allows us to view the future of this study with certain optimism.

- Limitations of the study: All the saliva samples obtained for the determination of salivary cortisol levels were collected in the morning (between 8 and 10 AM), exclusively detecting the level of morning salivary cortisol in all the participants. The number of patients with pathologies included was low, due to the low prevalence of the diseases studied and the use of strict exclusion criteria that limited the number of participants included. There is a need to carry out multicenter studies at an international level to increase the number of cases.

## Figures and Tables

**Table 1:** Baseline characteristics of the individuals under study according to group.

Characteristics	PMD	SCC	Control	*p*
	n =30	n =10	n =40	
Sex = Male (%)	15 (50.0)	5 (50.0)	17 (42.5)	0.798
Age (mean (SD))	60.43 (11.41)	64.70 (12.28)	56.95 (15.57)	0.240
Tobacco consumption = Yes (%)	10 (33.3)	8 (80.0)	18 (45.0)	0.037
Alcohol consumption = Yes (%)	9 (30.0)	4 (40.0)	19 (47.5)	0.335
Stress = Yes (%)	15 (50.0)	8 (80.0)	18 (45.0)	0.139

**Table 2:** Logistic regression model results for morning salivary cortisol level.

Variables	Coefficient	P Value	OR	OR - IC 95%	
				LL	UL
Intercept	1.02	0.079	2.77	0.93	9.33
SCC (Ref.: Control)	-1.42	0.205	0.24	0.02	1.88
PMD (Ref.: Control)	0.09	0.892	1.09	0.31	3.94
Tobacco consumption (Ref.: No)	-0.04	0.950	0.96	0.25	3.60
Alcohol consumption (Ref.: No)	1.98	0.005	7.21	1.94	33.05
Stress (Ref.: No)	-2.89	<0.001	0.06	0.01	0.18
